# Prevalence of Single and Multiple Natural NS3, NS5A and NS5B Resistance-Associated Substitutions in Hepatitis C Virus Genotypes 1–4 in Italy

**DOI:** 10.1038/s41598-018-26862-y

**Published:** 2018-06-12

**Authors:** Ada Bertoli, Maria Chiara Sorbo, Marianna Aragri, Ilaria Lenci, Elisabetta Teti, Ennio Polilli, Velia Chiara Di Maio, Laura Gianserra, Elisa Biliotti, Chiara Masetti, Carlo F. Magni, Sergio Babudieri, Laura A. Nicolini, Martina Milana, Pierluigi Cacciatore, Loredana Sarmati, Adriano Pellicelli, Stefania Paolucci, Antonio Craxì, Filomena Morisco, Valeria Pace Palitti, Massimo Siciliano, Nicola Coppola, Nerio Iapadre, Massimo Puoti, Giuliano Rizzardini, Gloria Taliani, Caterina Pasquazzi, Massimo Andreoni, Giustino Parruti, Mario Angelico, Carlo Federico Perno, Valeria Cento, Francesca Ceccherini-Silberstein, HCV Virology Italian Resistance Network (VIRONET-C), Pietro Andreone, Fausto Baldanti, Giorgio Barbarini, Vincenzo Boccaccio, Lucio Boglione, Matteo Bolis, Stefano Bonora, Vanni Borghi, Giuseppina Brancaccio, Savino Bruno, Bianca Bruzzone, Vincenza Calvaruso, Nicola Caporaso, Antonio Ciaccio, Alessia Ciancio, Piero Colombatto, Raffaele Cozzolongo, Cecilia D’Ambrosio, Gabriella D’Ettorre, Francesco De Leonardis, Andrea De Luca, Antonio Di Biagio, Giovanni Di Perri, Simona Francioso, Giovanni Battista Gaeta, Antonio Gasbarrini, Valeria Ghisetti, Alessia Giorgini, Antonio Grieco, Guido Gubertini, Roberto Gulminetti, Lara Lambiase, Simona Landonio, Miriam Lichtner, Ivana Maida, Simona Marenco, Letizia Marinaro, Renato Maserati, Michela Melis, Barbara Menzaghi, Elisa Meregalli, Valeria Micheli, Fosca Niero, Maurizio Paoloni, Alessandro Pieri, Maria Rendina, Dante Romagnoli, Barbara Rossetti, Tina Ruggiero, Vincenzo Sangiovanni, Mario Starace, Laura Sticchi, Pierluigi Tarquini, Pierluigi Toniutto, Vincenzo Vullo, Maurizio Zazzi

**Affiliations:** 1 Department Experimental Medicine and Surgery, University of Rome “Tor Vergata”, 00133 Rome, Italy; 2 Hepatology Unit, University Hospital of Rome “Tor Vergata”, 00133 Rome, Italy; 3 Infectious Diseases Unit, University Hospital of Rome “Tor Vergata”, 00133 Rome, Italy; 4 Infectious Diseases Unit, Pescara General Hospital, 65124 Pescara, Italy; 5 Infectious Diseases Unit, Sant’Andrea Hospital – “Sapienza” University, 00189 Rome, Italy; 6 Tropical Diseases, Umberto I Hospital –“Sapienza” University, 00161 Rome, Italy; 7 1st Division of Infectious Diseases, ASST Fatebenefratelli Sacco, 20157 Milan, Italy; 8 Clinical and Experimental Medicine, University of Sassari, 07100 Sassari, Italy; 9 Infectious Diseases Unit, IRCCS AOU San Martino - IST, 16132 Genoa, Italy; 10 Hepatology Unit, San Camillo Forlanini Hospital, 00151 Rome, Italy; 11 Molecular Virology, Fondazione IRCCS Policlinico San Matteo, 27100 Pavia, Italy; 12 Gastroenterology, “P. Giaccone” University Hospital, 90127 Palermo, Italy; 13 Gastroenterology, “Federico II” University, 80131 Naples, Italy; 14 Hepatology Unit, Pescara General Hospital, 65124 Pescara, Italy; 15 Gastroenterology, Catholic University of Rome, 00168 Rome, Italy; 16 Infectious Diseases Unit, “L. Vanvitelli” University of Campania, 80138 Naples, Italy; 17 Infectious Diseases Unit, S. Salvatore Hospital, 67100 L’Aquila, Italy; 18 Infectious Diseases Unit, Niguarda Ca’ Granda Hospital, 20162 Milan, Italy; 19 Haematology and Oncohematology, University of Milan, 20122 Milan, Italy; 20 Centro di Ricerca per lo Studio delle Epatiti, University of Bologna, 40138 Bologna, Italy; 21 Infectious and Tropical Diseases, Fondazione IRCCS Policlinico San Matteo, 27100 Pavia, Italy; 22 Humanitas University and IRCCS Istituto Clinico Humanitas, 20089 Rozzano, MI Italy; 23 Infectious Diseases Unit, Amedeo di Savoia Hospital, University of Turin, 10149 Turin, Italy; 24 Clinic of Infectious Diseases, AOUM, 41125 Modena, Italy; 25 Hygiene Unit, IRCCS AOU San Martino - IST, 16132 Genoa, Italy; 26 Center for Digestive Health, University of Milano-Bicocca, 20900 Monza, Italy; 27 Gastroenterology and Hepatology, University of Turin, 10126 Turin, Italy; 28 Hepatology Unit, AOUP, Ospedale Nuovo Santa Chiara, Cisanello, 56124 Pisa Italy; 29 Gastroenterology, “De Bellis” Hospital, IRCCS, 70013 Castellana Grotte, BA Italy; 30 Infectious Diseases Unit, “Sapienza” University of Rome, 00161 Rome, Italy; 31 Infectious Diseases Unit, Siena University Hospital, 53100 Siena, Italy; 32 Microbiology and Virology Laboratory, Amedeo di Savoia Hospital, 10126 Turin, Italy; 33 Unit San Paolo School of Medicine Department of Health Sciences, University of Milan, 20142 Milan, Italy; 34 Infectious Diseases Unit, Fondazione IRCCS Policlinico San Matteo, 27100 Pavia, Italy; 35 Infectious Diseases Unit, Sapienza University, 04100 Latina, Italy; 36 Gastroenterology Unit, IRCCS AOU San Martino - IST, 16132 Genoa, Italy; 37 Infectious Diseases Unit, Ospedale di circolo di Busto Arsizio, 21052 Busto Arsizio, VA Italy; 38 Clinical Microbiology, Virology and Bioemergencies, ASST Fatebenefratelli Sacco, 20157 Milan, Italy; 39 Infectious Disease Unit, Avezzano General Hospital, 67051 Avezzano, AQ Italy; 40 Gastroenterology Unit, University of Bari, 70121 Bari Bari, Italy; 41 Department of Biomedical, Metabolic and Neural Sciences, University of Modena and Reggio Emilia, 41125 Modena, Italy; 42 Third Infectious Disease Unit, A.O. Cotugno, 80131 Naples, Italy; 43 Infectious Disease Unit, Hospital “G. Mazzini”, 64100 Teramo, Italy; 44 Department of Experimental and Clinical Medicine, University of Udine, 33100 Udine, Italy; 45 Department of Medical Biotechnology, University of Siena, 53100 Siena, Italy

## Abstract

Natural resistance-associated substitutions (RASs) are reported with highly variable prevalence across different HCV genotypes (GTs). Frequency of natural RASs in a large Italian real-life cohort of patients infected with the 4 main HCV-GTs was investigated. NS3, NS5A and NS5B sequences were analysed in 1445 HCV-infected DAA-naïve patients. Sanger-sequencing was performed by home-made protocols on 464 GT1a, 585 GT1b, 92 GT2c, 199 GT3a, 16 GT4a and 99 GT4d samples. Overall, 20.7% (301/1455) of patients showed natural RASs, and the prevalence of multiclass-resistance was 7.3% (29/372 patients analysed). NS3-RASs were particularly common in GT1a and GT1b (45.2-10.8%, respectively), mainly due to 80K presence in GT1a (17%). Almost all GTs showed high prevalence of NS5A-RASs (range: 10.2–45.4%), and especially of 93H (5.1%). NS5A-RASs with fold-change >100x were detected in 6.8% GT1a (30H/R-31M-93C/H), 10.3% GT1b (31V-93H), 28.4% GT2c (28C-31M-93H), 8.5% GT3a (30K-93H), 45.5% GT4a (28M-30R-93H) and 3.8% GT4d (28V-30S-93H). Sofosbuvir RAS 282T was never detected, while the 159F and 316N RASs were found in GT1b (13.4–19.1%, respectively). Natural RASs are common in Italian patients infected with HCV-GTs 1–4. High prevalence of clinically-relevant RASs (such as Y93H) supports the appropriateness of HCV resistance-test to properly guide DAA-based therapy.

## Introduction

In the last few years, the management of chronic Hepatitis C virus (HCV) infection was revolutioned by the introduction of new anti-HCV therapies based on direct-acting antiviral agents (DAAs). Short, highly effective and well tolerated interferon-free combinations of DAAs targeting three distinct non-structural HCV proteins (NS3–4A protease, NS5A, and NS5B polymerase) are now available for clinical use, allowing to reach outstanding cure rates even in patients that once were considered “difficult-to-treat”^1^.

Nevertheless, viral failure can still occur in a 5–10% of patients, most of which present clinical and virological characteristics that, when combined, can reduce DAA efficacy^2–4^. Among virological ones, NS3 and (more importantly) NS5A substitutions naturally present in the infecting viral strain were shown to significantly reduce sustained viral response (SVR) rates to NS3-protease inhibitors (PI) and NS5A-inhibitors^5–9^.

Natural presence of RASs is a unique characteristic of HCV, not shared with other viruses responsible for chronic infections, such as HBV or HIV. It depends on the extremely high intrinsic genetic variability of HCV, consequence of its high mutational rate and replication turn-over, along with lack of proof-reading activity by viral polymerase.

Natural RASs are reported with highly variable prevalence across different HCV genotypes (GTs)^10^ but some of them, when present, are able to significantly reduce SVR rates to specific DAAs in complex patients (infection by HCV GT1a or 3, high baseline viremia, previous treatment experience, cirrhosis presence)^1,5,9,11,12^. Even with newer DAAs, for instance, patients with GT3 infection and natural RASs may more frequently experience virological failure^13,14^. Under these conditions, a tailored approach in some (not infrequent, though) real-life situations may help in bringing the cure rate closer to the 98–99%^5,9,11,15^.

Thanks to new and highly effective DAAs, HCV treatment is moving towards simpler regimens, the chances to use proficiency very short and ribavirin-free regimens will probably rely on a pre-selection of eligible patients, a selection that could likely take into account also natural resistance. So far, the natural prevalence of RASs was not extensively defined for some populations, such as non GT1 infected patients, patients with cirrhosis, and/or patients with previous IFN experience. In addition, very few reports on circulation of HCV GTs and subtypes in Italy are present in literature to date, as large dataset of HCV sequences were not available. A recent review of HCV GTs distribution in selected West European regions from 2011–2015 estimated, in Italy, a 62% prevalence of GT1 infection (21.8% 1a, 37.4% 1b, 2.8% not specified), 15.4% GT2, 14.9% GT3, 7.6% GT4 and 0.1% GT5^16^.

The aim of this study was to extensively characterize the presence of natural RASs in the NS3, NS5A and NS5B regions of HCV, in a large clinical multicentre database from real-life clinical practice in DAA naïve patients, able to represent the main HCV GTs and subtypes circulating in Italy.

## Results

### Study population

Natural RASs were analysed in 2618 sequences from 1455 chronic HCV-infected patients naïve to NS3- (N = 1032), and/or NS5A- (N = 1090), and/or NS5B-inhibitors (N = 496).

Clinical and virological characteristics of the 1455 patients included are summarized in Table 1. The patients were aged 56 (50–66) years, and 69.6% were male; 489/1455 (33.6%) patients were IFN-naïve. Half of patients (55%) had a liver cirrhosis, and a minority had a history of liver transplant (4.5%) or hepatocellular carcinoma (HCC, 4.1%). Fibroscan value and/or Metavir score was available for 1279/1455 patients included. Of them 750 (58.6%) had F4-cirrhosis. A small numer of patients had F0/F1 fibrosis (N = 22, 1.7%) or F2 fibrosis (N = 146, 11.4%), while 361 had F3 fibrosis (N = 361, 28.2%). Child-Pugh was available for 213 cirrhotic patients, and of them only 28 had decompensated cirrhosis.

**Table 1:** Demographic characteristics of the study population.

		HCV genotype and subtype						Overall
		1a	1b	2c	3a	4a	4d	
Patients included, N		464	585	92	199	16	99	1455
Males, N(%)		381 (82.1)	317 (54.2)	51 (55.4)	171 (85.9)	14 (87.5)	79 (79.8)	1013 (69.6)
Age (years), Median (IQR)		52 (47–57)	65 (55–72)	70 (63–76)	54 (49–58)	53 (43–56)	53 (49–57)	56 (50–66)
BMI (Kg/m^2^), Median (IQR)		25 (22–29)	26 (24–30)	28 (23–34)	24 (22–27)	27 (27–27)	24 (22–26)	25 (23–28)
IL-28B genotype^a^	CC	31 (22.0)	19 (9.7)	1 (12.5)	10 (38.5)	1 (33.3)	2 (9.1)	64 (16.2)
	CT	91 (64.5)	125 (64.1)	5 (62.5)	12 (46.2)	2 (66.7)	14 (63.6)	249 (63.0)
	TT	19 (13.5)	51 (26.2)	2 (25.0)	4 (15.4)	0 (0.0)	6 (27.3)	82 (20.8)
Cirrhosis, N(%)		214 (46.1)	347 (59.3)	48 (52.2)	118 (59.3)	8 (50)	65 (65.7)	800 (55)
Liver transplant, N(%)		15 (3.2)	28 (4.8)	2 (2.2)	16 (8.0)	1 (6.3)	3 (3.0)	65 (4.5)
HCC, N(%)		4 (0.9)	35 (6.0)	7 (7.6)	10 (5.0)	0 (0.0)	3 (3.0)	59 (4.1)
IFN naive, N(%)		175 (37.7)	160 (27.4)	46 (50)	79 (39.7)	5 (31.3)	24 (24.2)	489 (33.6)
HBV coinfection, N(%)		19 (4.1)	10 (1.7)	0 (0.0)	9 (4.5)	1 (6.3)	0 (0.0)	39 (2.7)
HIV coinfection, N(%)		36 (7.8)	6 (1.0)	1 (1.1)	19 (9.5)	0 (0.0)	14 (14.1)	76 (5.2)

^a^Available for 141 GT-1a, 195 GT-1b, 8 GT-2c, 26 GT-3a, 3 GT-4a and 22 GT-4d patients. IQR, interquartile range; BMI, body mass index; HCC, hepatocellular carcinoma; IFN, interferon.

Patients with HIV and HBV coinfection were 5.2% (76/1455) and 2.7% (39/1455), respectively.

All HCV-GTs circulating in Europe were well represented, since we included 464 GT1a, 585 GT1b, 92 GT2c, 199 GT3a, 16 GT4a and 99 GT4d samples. In addition, clinical centres collaborating in enrolling patients were distributed across all the Italian territory, from North to South and including also Sardinia and Sicily (see Supplementary Fig. S1).

The overall prevalence of natural RASs in at least 1 DAA target was 20.7% (301/1455).

### Prevalence of natural NS3, NS5A and NS5B RASs

At least 1 NS3-RAS was detected in 211/1032 patients analyzed (20.4%). In particular, 97/1032 (9.4%) presented RASs able to confer low-level of resistance (fold-change < 100), and 6/1032 (0.6%) had RASs associated with intermediate levels of resistance (fold-change 100–1000) (Table 2).

**Table 2:** Natural NS3 resistance associated substitutions detected in patients naïve to NS3-protease inhibitors.

		Natural NS3 RASs prevalence among HCV genotypes						
		1a, N = 336	1b, N = 427	2c, N = 62	3a, N = 130	4a, N = 11	4d, N = 66	Overall, N = 1032
Patients with at least 1 RAS, N (%)^a^	*Overall*	152 (45.2)	46 (10.8)	2 (3.2)	6 (4.6)	1 (9.1)	4 (6.1)	211 (20.4)
	*Low-level RAS*	74 (22.0)	15 (3.5)	2 (3.2)	4 (3.1)	0 (0.0)	2 (3.0)	97 (9.4)
	*Intermediate-level RAS*	1 (0.3)	3 (0.7)	0 (0.0)	1 (0.8)	0 (0.0)	1 (1.5)	6 (0.6)
**Specific RASs at NS3 positions, N(%)**								
36	V36L	*14 (4.2)*	*8 (1.9)*		*1 (0.8)*			*23 (2.2)*
	V36M	4 (1.2)						4 (0.4)
43	F43L				1 (0.8)			1 (0.1)
54	T54A	*1 (0.3)*						*1 (0.1)*
	T54S	*12 (3.6)*	*8 (1.9)*		*1 (0.8)*	*1 (9.1)*	*2 (3.0)*	*24 (2.3)*
55	V55A	*13 (3.9)*	*2 (0.5)*					*15 (1.5)*
	V55I	*8 (2.4)*	*1 (0.2)*		*1 (0.8)*			*10 (1.0)*
56	Y56H		1 (0.2)					1 (0.1)
80	Q80K	58 (17.3)	5 (1.2)		2 (1.5)			65 (6.3)
	Q80R	2 (0.6)	2 (0.5)					4 (0.4)
122	S122R	1 (0.3)						1 (0.1)
155	R155K	**1 (0.3)**						**1 (0.1)**
156	A156G		1 (0.2)					1 (0.1)
	A156S		1 (0.2)					1 (0.1)
168	D168A		**1 (0.2)**					**1 (0.1)**
	D168E	2 (0.6)	3 (0.7)	2 (3.2)			2 (3.0)	9 (0.9)
	D168Q				**1 (0.8)**			**1 (0.1)**
	D168T	1 (0.3)						1 (0.1)
	D168V		**2 (0.5)**				**1 (1.5)**	**3 (0.3)**
170	V170A		2 (0.5)					2 (0.2)

^a^NS3 amino acid substitutions were classified according to the *in vitro* fold-change reduction in protease inhibitors activity in the specific HCV genotype. Low-level RASs (fold-change 2–100) are reported as plain text, while intermediate-level RASs (fold-change 100–1000) are reported in **bold**. RASs observed *in vivo* or proposed to be associated with resistance (no fold-change available) are reported in *italics*. RAS, resistance-associated substitution.

**Figure 1:**
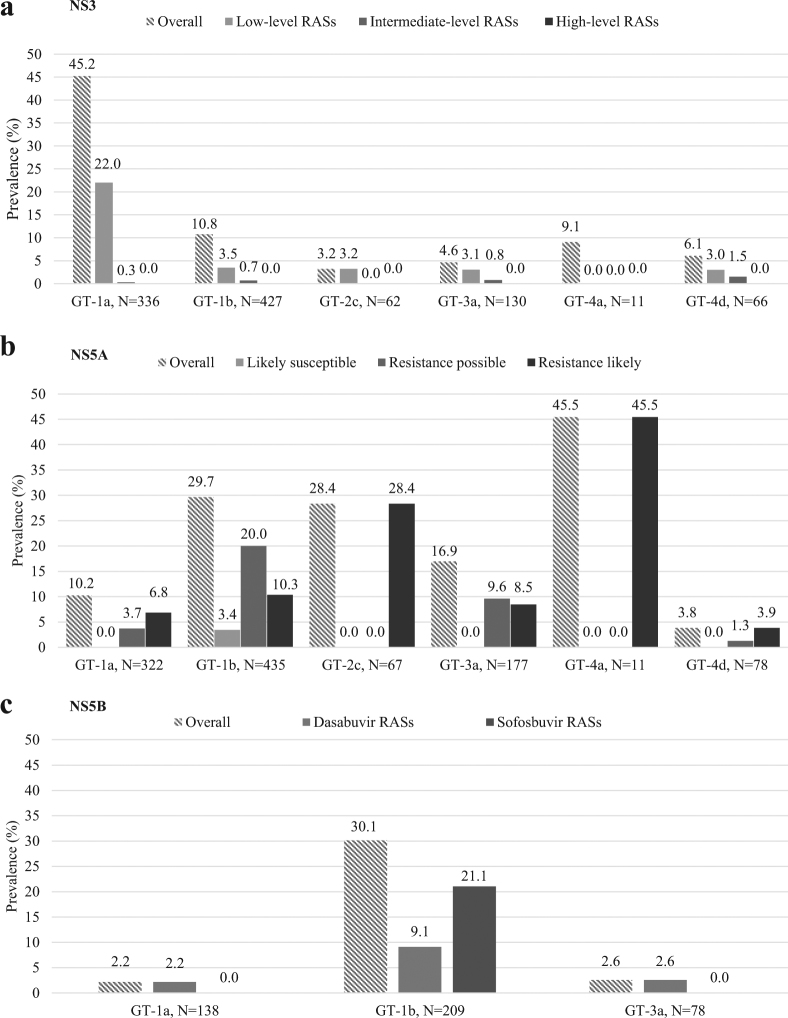
Prevalence of natural NS3, NS5A and NS5B resistance-associated substitutions by HCV genotype and subtype. NS3 resistance-associated substitutions (RASs) in **panel a** were classified according to the *in vitro* fold-change reduction in protease inhibitors activity in the specific HCV genotype. Low-level RASs were defined by fold-changes between 2 and 100, while intermediate-level RASs were defined by fold-change between 100 and 1000. The overall prevalence also include RASs observed *in vivo* or proposed to be associated with resistance (no fold-change available). NS5A substitutions in **panel b** were divided both according to the *in vitro* fold-change reduction, and to their potential association with resistance in *vivo*. For 1st generation NS5A-inhibitors, RASs with fold-change 2.5–20× are reported as “likely susceptible”; RASs with fold-change 20–100 or only *in vivo* RAS (no fold-change available) are reported as “resistance possible”. Lastly, RASs with fold-change >100× are defined as “resistance likely”. For 2nd generation NS5A-inhibitors elbasvir, pibrentasvir and velpatasvir, RASs with fold-change <2.5× are reported as “likely susceptible”, RASs with fold-change 2.5–9 or only *in vivo* RAS (no fold-change available) are reported as “resistance possible”. Lastly, RASs with fold-change >10x are reported as “resistance likely”. Additional rule applied: 1 level up if found in virologic failure. NS5B substitutions in **panel c** were reported separately for dasabuvir or sofosbuvir. RAS, resistance-associated substitution.

Natural NS3 RASs were mainly present in GT1a (152/336, 45.2%) and GT1b (46/427, 10.8%) (Fig. 1, panel A), with high prevalence of Q80K in GT1a (58/336, 17%). The Q80K was never found in GT4. Intermediate/high level RASs D168A/E/T/V were found with a prevalence of 3% (2/62) in GT2c, and of 4.5% (3/66) in GT4d (Table 2).

Prevalence of NS3 RASs did not significantly differ according to previous treatment (IFN-failures vs. naïve), nor presence of cirrhosis (see Supplementary Fig. S2).

Natural NS5A RASs were found in 219/1090 (20.1%) patients, with higher prevalence in GT1a (33/322, 10.2%), GT1b (129/435, 29.7%), GT2c (19/67, 28.3%), GT3a (30/177, 16.9%), GT4a (5/11, 45.4%); in GT4d the prevalence was low (3/78, 3.8%) (Fig. 1, panel B; and Table 3). Likely resistant NS5A substitutions were detected mainly in GT1b (10.3% prevalence [45/435]; L31V and Y93H), in GT2c (28.4% prevalence [19/67]; F28C, L31M, and Y93H), and in GT4a (45.5% prevalence [5/11]; V28M, L30R, and Y93H), while were detected with a frequency of 6.8% (22/322) in GT1a (Q30H/R, L31M, Y93C/H), of 8.5% (15/177) in GT3a (A30K, Y93H), and of 3.8% (3/78) in GT4d (M28V, R30S, Y93H).

**Table 3:** Natural NS5A resistance associated substitutions detected in patients naïve to NS5A inhibitors.

		Natural NS5A RASs prevalence among HCV genotypes						
		1a, N = 322	1b, N = 435	2c, N = 67	3a, N = 177	4a, N = 11	4d, N = 78	Overall, N = 1090
Patients with at least 1 RAS, N(%)^a^	***Overall***	33 (10.2)	129 (29.7)	19 (28.4)	30 (16.9)	5 (45.5)	3 (3.8)	219 (20.1)
	*Resistance likely*	22 (6.8)	45 (10.3)	19 (28.4)	15 (8.5)	5 (45.5)	3 (3.8)	109 (10.0)
	*Resistance possible*	12 (3.7)	87 (20.0)	0 (0.0)	17 (9.6)	0 (0.0)	1 (1.3)	117 (10.7)
	*Likely susceptible*	0 (0.0)	15 (3.4)	0 (0.0)	0 (0.0)	0 (0.0)	0 (0.0)	15 (1.4)
Patients with >1 RAS, N(%)^a^	***Overall***	2 (0.6)	21 (4.8)	2 (3.0)	3 (1.7)	2 (18.2)	1 (1.3)	31 (2.8)
	*Resistance likely*	1 (0.3)	1 (0.2)	2 (3.0)	1 (0.6)	2 (18.2)	0 (0.0)	7 (0.6)
**Specific RASs at NS5A positions**								
24	*K24R*	**1 (0.3)**						1 (0.1)
	*Q24K*		**4 (0.9)**					4 (0.4)
28	*F28C*			**17 (25.4)**				17 (1.6)
	*L/M28V*	**11 (3.4)**					**1 (1.3)**	12 (1.1)
	*L/V28M*		15 (3.4)			**3 (27.3)**		18 (1.7)
30	*Q30H*	**2 (0.6)**						2 (0.2)
	*A30K*				**7 (4.0)**			7 (0.6)
	*Q/L30R*	**6 (1.9)**				**3 (27.3)**		9 (0.8)
	*R30Q*		**22 (5.1)**					22 (2.0)
	*R30S*						**1 (1.3)**	1 (0.1)
	*A30V*				**1 (0.6)**			1 (0.1)
31	*L31I*		**2 (0.5)**					2 (0.2)
	*L31F*				**1 (0.6)**			1 (0.1)
	*L31M*	**9 (2.8)**	**21 (4.8)**	**3 (4.5)**				33 (3)
	*L31P*				**1 (0.6)**			1 (0.1)
	*L/M31V*		**2 (0.5)**				**1 (1.3)**	3 (0.3)
58	*P58A*		**1 (0.2)**					1 (0.1)
	*P58L*		**3 (0.7)**					3 (0.3)
	*P58S*		**15 (3.4)**					15 (1.4)
	*P58T*		**4 (0.9)**					4 (0.4)
62	*A62L*				**15 (8.5)**			15 (1.4)
92	*A92T*		**20 (4.6)**					20 (1.8)
**93**	*Y93C*	**2 (0.6)**	**1 (0.2)**					3 (0.3)
	*Y93H*	**1 (0.3)**	**44 (10.1)**	**1 (1.5)**	**8 (4.5)**	**1 (9.1)**	**1 (1.3)**	56 (5.1)
	*Y93I*		**1 (0.2)**					1 (0.1)
	*Y93N*	**3 (0.9)**						3 (0.3)

^a^NS5A amino acid substitutions were classified according to the *in vitro* fold-change reduction in protease inhibitors activity in the specific HCV genotype. For 1st generation NS5A-inhibitors, RASs with fold-change >100× are reported in bold and underlined (**resistance likely**); RASs with fold-change 20–100 or only *in vivo* RAS, no fold-change, are reported in bold (**resistance possible**); RASs with fold-change 2.5–20× are reported plain text (likely susceptible). For 2nd generation NS5A-inhibitors elbasvir, pibrentasvir and velpatasvir, RASs with fold-change >10× are reported in bold and underlined (**resistance likely**); RASs with fold-change 2.5–9 or only *in vivo* RAS, no fold-change, are reported in bold (**resistance possible**); RASs with fold-change <2.5× are reported in plain text (likely susceptible). Additional rule applied: 1 level up if found in virologic failure. RAS, resistance-associated substitution.

The most common NS5A RAS across all genotypes was Y93H, with an overall prevalence of 5.1% (56/1090). Its prevalence was particularly high in GT1b (44/435, 10.1%) (Table 3).

Of the 1090 patients analyzed, 31 (2.8%) presented multiple NS5A RASs (Table 3). The contemporaneous detection of >1 NS5A RASs was more common in GT1b patients (21/435, 4.8%) and GT4a (2/11, 18.2%), but only few patients had >1 likely resistant NS5A RASs.

Prevalence of NS5A RASs did not significantly differ according to presence of cirrhosis, nor to treatment experience, (see Supplementary Fig. S3).

Among the 496 patients evaluated, natural NS5B RASs were detected exclusively in GT1a, GT1b and GT3a (Fig. 1, panel C; and Table 4).

**Table 4:** Natural NS5B resistance associated substitutions detected in patients naïve to NS5B non nucleoside and nucleotide inhibitors.

	Fold-change	Natural NS5B RASs prevalence among HCV genotypes, N (%)			
		1a, N = 138	1b, N = 209	3a, N = 78	Overall, **N** = **496**
***Dasabuvir RASs***					
Patients with at least 1 RAS, N(%)		3 (2.2)	19 (9.1)	2 (2.6)	24 (4.8)
**S368T**	*100–1000*	2 (1.4)			2 (0.4)
**M414I**	*100–1000*	1 (0.7)	1 (0.5)		2 (0.4)
E446K	*<100*			1 (1.3)	1 (0.2)
**Y448C**	*100–1000*			1 (1.3)	1 (0.2)
S556G	*<100*		18 (8.6)		18 (3.6)
***Sofosbuvir RASs***					
Patients with at least 1 RAS, N(%)			44 (21.1)		44 (8.9)
L159F	*<100*		28 (13.4)		28 (5.6)
C316H	*n.a*.		1 (0.5)		1 (0.2)
C316N	*n.a*.		40 (19.1)		40 (8.1)
V321A	*<100*		1 (0.5)		1 (0.2)

Mutations with >100 fold-change are reported in bold; n.a. as not available. RAS, resistance-associated substitution.

GT1b showed the higher prevalence of NS5B RASs (30.1%, 63/209) (Fig. 1, panel C), mainly due to frequent presence of L159F and C316N polymorphisms (13.4% and 19.1% prevalence, respectively). These two polymorphisms were usually found together, as also confirmed by covariation analysis (phy = 0.67, P < 0.001). The prevalence of both L159F and C316N tended to be higher in IFN-experienced GT1b patients (14.4% and 20.9% respectively), than in IFN-naïve (10.5% and 14.0% respectively), though the difference was not statistically significant (P = 0.64 and P = 0.32 by Fisher exact test, respectively).

### Prevalence of multiple natural RASs on different DAAs gene targets

Among the 372 patients who were tested for natural resistance on all 3 genes, 29 (7.3%) showed multiple RASs on >2 drug-targets. The most prevalent association was of NS3 plus NS5A RASs (10/372, 2.7%), followed by NS3 plus NS5B RASs (7/372, 1.9%), NS5A plus NS5B RASs (6/372, 1.6%) and NS5B-NI (nucleoside NS5B polymerase inhibitors) plus NS5B-NNI (non-nucleoside NS5B polymerase inhibitors) RASs (4/372, 1.1%, all GT1b) (see Supplementary Fig. S4).

Multiple RASs on 3 drug-targets were detected exclusively in 2 GT1b patients (0.5%).

Covariation analysis revealed a cross-target association of RASs only in patients with GT1 infection. In GT1b, NS5A-P58S and NS5B-V321A RASs were significantly associated (phy = 0.44, P = 0.03 by covariation analysis), as well as NS3-S122A and NS5A-M28V RASs in GT1a (phy = 0.40, P = 0.04 by covariation analysis) (see Supplementary Fig. S5).

## Discussion

Thanks to a nation-wide collaboration (Vironet C), the present study collects a large number of sequences from DAA-naïve patients, able to represent the circulation of HCV resistant strains in Italy. Among the 1455 patients included in the analysis, 20.7% were infected with viral strains harboring at least one RASs in one of the 3 DAA-targeted regions.

Across all GTs, NS5A RASs were the most frequently detected (20.1%, 219/1090), especially in GT1b (29.7%, 129/435). A previous global study reported a much higher prevalence of RASs in GT1b (41.7%)^17^, but our results are concordant with a recent Italian study, that highlighted natural NS5A RASs in 23% (14/61) GT1b patients^18^. A smaller Italian local dataset also reported a 11.9% natural NS5A-RASs prevalence in 45 GT3 patients^19^, slightly lower than the 16.9% we found in our 177 GT3 patients.

Notably, when the analysis was limited to amino acid substitutions associated with a clinically relevant level or resistance (defined as “likely resistant”), their prevalence dropped to 6.8% in GT1a, 10.3% in GT1b and 8.5% in GT3a. In addition, “likely resistant” substitutions were found with high prevalence in GT2c patients (28.4%, mainly due to F28C presence), and GT4a (45.5% for L30R and V28M presence). To our knowledge, F28C essentially affects the efficacy of daclatasvir in GT2a replicons *in vitro* (FC = 400)^20^, but no data are available for F28C in GT2c patients treated with daclatasvir or velpatasvir. However, it is interesting to note that in Italy the prevalence of F28C in DAA naïve population (25.4%) is much lower than that found in NS5A-failing GT2c patients (66.7%, 4/6; p = 0.05)^21^. Since GT2 patients are generally treated with short, RBV-free regimens, the role of F28C in treatment failure will probably deserve further attention.

Much better defined is the role of other “likely resistant” viral variants, such as those harboring substitutions at NS5A positions 93 and 30. In our population, the Y93H was far the most common natural NS5A-RASs detected, with an overall prevalence of 5.1% (56/1090), but reaching 10.1% prevalence in GT1b and 9.1% in GT4a. Recently, this RASs has been reported with very high prevalence also in other “rare” GT4 subtypes, such as GT4b (50%) and GT4r (13%)^10^.

The Y93H was associated with a significant drop in DAA efficacy in some HCV GTs, such as GT1a and GT3 and clinical conditions, as presence of cirrhosis^11,22,23^. For instance, even though tested on a very small number of patients, the Y93H presence in GT3 IFN-experienced, DAA naïve patients reduced by 50% the efficacy of 12-weeks glecaprevir/pibrentasvir treatment^22^. In the present study, Y93H was rarely detected in both GT1a (0.3%) and GT3a (4.5%). This result reflects that of a recent global analysis of natural HCV RASs in 7893 subjects, where the Y93H was found with a prevalence of 11% in GT-1b and 6% in GT3a, while it was never detected in GT1a^10^.

In clinical trials, the presence of multiple natural NS5A RASs was associated with a further reduction in treatment efficacy as compared to single RASs^11^. The detection of multiple NS5A RASs in NS5A-naïve patients is quite uncommon. In our population, only 2.8% of patients (31/1090) had more than one natural NS5A RAS, and 0.6% (7/1090) had “likely resistant” RASs. Multiple RASs detection was more common in GT1b (4.8%), a GT considered easy-to-treat and thus not usually investigated for natural RASs and preferentially treated with short and RBV-free regimens. Nevertheless, no data are available on a possible impact of multiple NS5A RASs in treatment efficacy for GT1b infected patients, and results obtained in GT1 up to now comes from a limited number of patients, and could be confounded by the presence of RASs with major impact even in the absence of other NS5A polymorphisms^11^. In addition, as full-length sequencing was not performed in our study, we cannot drawn the conclusion that individual amplicons of each region actually belong to the same virus^24^.

NS3 RASs were also common (20.4%, 211/1032), particularly due to their high prevalence in GT1a (45.2%, 152/336), concordant with previous literature data^17,25^, and consequence of the frequent detection of Q80K RAS (17.3% in GT1a, 6.3% overall). Much less frequent were natural RASs at position 168, always found with a prevalence <5%.

Only few patients harbored specific NS5B RASs for sofosbuvir (8.9%) or dasabuvir (4.8%). As expected, the major RAS for sofosbuvir S282T was never detected^9^, while the NS5B putative RASs L159F, C316N and S556G were found in 13.4%, 19.1% and 8.6% GT1b patients respectively, often in association. Literature data on these 3 RASs report very low fold-change values for sofosbuvir and dasabuvir^26,27^, however they have been described with higher prevalence in failing patients^3,21,28–31^, and therefore their impact is not yet completely understood.

Along with HCV sequence data, we were also able to collect clinical information, such as treatment history and liver status, that allowed a more in-depth characterization of the phenomenon of natural HCV drug resistance. Overall, we found no significant associations among presence or absence of cirrhosis and prevalence of natural RASs. Similarly previous treatment with IFN-based regimens was not significantly correlated with higher RASs prevalence in any of the HCV-GT analyzed. However, we noticed that both L159F and C316N were more frequently detected in interferon/ribavirin-experienced (7% and 11% respectively) than in naïve patients (3% and 4% respectively). The reason of this different prevalence, though not statistically significant, is unclear. Ribavirin is a guanosine analogue that, among the proposed mechanism of action, may directly inhibit NS5B polymerase^32^ and/or promote mutagenesis^33^, thus potentially contribute to explain the more frequent generation and then selection of these substitutions in particular conditions (such as in GT1b). However, no experimental model of viral relapse with ribavirin are available, and the lack of clear data on ribavirin resistance makes this hypothesis difficult to further support.

This is one of the few studies, to our knowledge, able to assess the prevalence of patients showing contemporaneous presence of multiple RASs on more than 1 DAA target. Indeed, by analyzing within the 4 main HCV-GT circulating in Italy, we found multiclass resistance in 7.3% of patients, most frequently in GT-1b, represented by a combination of RASs in NS3 and in NS5A. Bartels *et al*. already analyzed the combinations of natural resistant variants across NS3, NS5A, and NS5B in GT1, and in their study they were rare, and most combinations were observed in a single patient^34^.

While the role of single-class NS3 or NS5A RASs has been discussed quite extensively, the role of multiclass resistance is still to be defined. Among DAAs combinations currently approved for clinical use, or soon to be, several involve a combination of NS3 and NS5A-inhibitors^1,35–38^. The excellent efficacy of these combinations in DAA-naïve patients makes difficult to interpret the role of multiclass resistance, even though the analysis of the few patients who failed to achieve HCV cure, especially with shorter regimens, may suggest a potential impact of double NS3 and NS5A class resistance. For instance, natural NS3 + NS5A RASs were recently observed in treatment-experienced GT3 patients who failed glecaprevir/pibrentasvir 12-weeks regimen^22^, and the presence of natural RASs specifically relevant for this genotype (i.e. A30K and Y93H in NS5A, ± NS3 polymorphisms) in this category of patients would probably require a prolongation of DAAs treatment duration to 16 weeks^22^. Similar results were highlighted in NS5A-experienced patients, in whom the presence of double-class resistance for glecaprevir/pibrentasvir is undoubtedly much relevant, and the impact of retreatment efficacy is higher^39,40^.

In conclusion, our study contributed to a better definition of the presence and circulation of resistant viral strains in a large Italian population of patients infected with HCV-GTs 1–4. We were able to uncover natural RASs presence across all HCV-GT analyzed, highlighting multiple NS5A-RASs presence in 2.8% of patients, and multiclass resistance in 7.3%. Clinical application of these findings remains challenging, as genotypic resistance testing is not available everywhere, and prescription of DAA is often limited by costs and reimbursement policies. Nevertheless, especially in countries with high prevalence of HCV infection and where RAS testing is available and validated, the knowledge of natural RASs burden in specific HCV GTs and subtypes may help focusing HCV diagnostic interventions in clinical conditions and settings that would maximize DAA efficacy rate, potentially avoiding wrong and expensive therapies.

## Methods

### Patients

This is an Italian multicentre, observational study involving DAA-naïve patients with chronic HCV-infection, recruited from 23 Italian clinical centres between 2011 and 2016. A detailed description of Italian regions involved is included in Supplementary Fig. S1.

Eligible patients were DAA-naive who had at least one HCV genotypic resistance testing (GRT) performed by population sequencing on any target region (NS3, NS5A and/or NS5B) for routine clinical purposes or for research. Approval by ethics committee was deemed unnecessary for all patients evaluated for diagnostic purpose according to Italian law, because this was not an hypothesis of clinical trial on medicinal products for clinical use (art. 6 and art. 9, leg. decree 211/2003). In the cases evaluated only for research purpose, approval by the local Ethics Committees and patient written informed consent were obtained. All samples used for HCV sequencing, either for clinical or only research purpose, were all anonymously collected and analyzed according to Italian law (leg. decree 196/2003). This study was conducted in accordance with the principles of the Declaration of Helsinki. All information, including virological, clinical, and therapy data, were recorded in an anonymous database.

### HCV Sanger sequencing

Sanger sequencing of the NS3-protease (aa 1–181), NS5A domain I (aa 1–213) and NS5B (aa 1–591) proteins was performed in plasma samples by using home-made protocols specific for each HCV genotype/subtype in 4 Italian laboratories. Overall, 93% of sequences were obtained in Tor Vergata laboratory, and all laboratories who contributed with HCV sequences to the present study, recently participated to a nationwide validation program for HCV GRT, in which concordance in mutation detection and report was > 85% in all 3 genes^41^, thus making the possible discordance of sequencing methods marginally relevant to the present study. Detailed sequencing procedure is reported elsewhere^21,42–45^.

### HCV resistance mutation analysis

Wild-type amino acids were defined according to references sequences from Geno2Pheno tool^46^.

The list of amino acid substitutions reported to confer reduced susceptibility (>2-fold increased EC_50_) to currently and soon-to-be available DAAs was defined according to published data, that also allowed the interpretation and classification of clinical impact of RASs^47^. Details are as follows.

NS3 and NS5B RASs were classified according to fold-change reduction levels in different HCV-genotypes. Low-level resistance: fold-change 2–100; Intermediate-level resistance: fold-change 100–1000; High-level resistance: fold-change >1000.

NS5A RASs were classified both according to the *in vitro* fold-change reduction, and to their potential association with resistance in *vivo*. For 1st generation NS5A-inhibitors, RASs with fold-change >100× are defined as resistance likely; RASs with fold-change 20–100 or only *in vivo* RAS (no fold-change) are defined as resistance possible; RASs with fold-change 2.5–20× are defined as likely susceptible. For 2nd generation NS5A-inhibitors elbasvir, pibrentasvir and velpatasvir, RASs with fold-change >10× are defined as resistance likely; RASs with fold-change 2.5–9 or only *in vivo* RAS (no fold-change), are defined as resistance possible; RASs with fold-change <2.5× are defined as likely susceptible. If RASs were found *in vivo* at virological failure, the level of resistance was increased by 1 level.

The criteria to be included in the present analysis as in vivo RASs were: a) to be found as de novo developed variant in failing patients in literature; or b) to have a demonstrated impact on virological response even if found as natural RAS in literature.

### Phylogenetic analysis of NS3, NS5A and NS5B sequences

Phylogenetic analysis was performed to check the possibility of cross-contamination or sample mix-up during laboratory procedures, and also to evaluate concordance with previous subtype assignment by commercial genotyping assays.

NS3-protease, NS5A or NS5B sequences were aligned using Clustal W algorithm integrated into the BioEdit software. Then, all sequences were compared with reference strains of genotype 1 to 7 (GeneBank accession numbers: HCV-1a, M62321; HCV-1b, D90208; HCV-2a, D10988; HCV-2c, D50409; HCV-3a; HCV-4a, Y11604; HCV-4d, FJ462437, the same proposed by Geno2Pheno tool^46^) using the neighbor-joining method and the Kimura two-parameter distance estimation approach in MEGA v7^48^. The reliability of the phylogenetic clustering was evaluated using bootstrap analysis with 1000 replicates. The significance of the group was assumed when bootstrap values were >70%.

### Statistical analysis

Results are expressed as median values and interquartile range (IQR) for continuous data and number (percentage) for categorical data. Categorical variables were compared using the Chi-squared test and fisher’s exact test when was appropriate. All the analyses were performed using the SPSS software package (version 23.0) for Windows (SPSS Inc., Chicago, IL).

### Mutation covariation and cluster analysis

Binomial-correlation coefficient (phi) was calculated to assess covariation among RASs, either on the same or on different genomic regions. Statistically significant pairs of RASs were identified by Fisher’s exact test, and then corrected for multiple-testing by Benjamini–Hochberg method (FDR = 0.05).

In order to identify and summarize higher-order interactions of RASs, we transformed the pairwise phi correlation coefficients into dissimilarity values. A dendrogram was then computed by hierarchical clustering, and its stability was assessed from 100 bootstrap replicates.

All analyses were performed in R software.

The details of this explorative data analysis procedure have been described elsewhere^49^.

## Electronic supplementary material

Supplementary Figures
