# Supplementary material for: Prevalence of Single and Multiple Natural NS3, NS5A and NS5B Resistance-Associated Substitutions in Hepatitis C Virus Genotypes 1–4 in Italy
Source: Sci Rep. 2018 Jun 12;8:8988. doi: 10.1038/s41598-018-26862-y (PMC5997636; doi:10.1038/s41598-018-26862-y)
Supplement: Supplementary file 1 — Supplementary Figures [file 41598_2018_26862_MOESM1_ESM.pdf]

SUPPLEMENTARY INFORMATION

Prevalence of Single and Multiple Natural NS3, NS5A and NS5B Resistance-Associated Substitutions in Hepatitis C Virus Genotypes 1-4 in Italy

Ada Bertoli[1], Maria Chiara Sorbo[1], Marianna Aragri[1], Ilaria Lenci[2], Elisabetta Teti[3], Ennio Polilli[4], Velia Chiara Di Maio[1], Laura Gianserra[5], Elisa Biliotti[6], Chiara Masetti[2], Carlo F. Magni[7], Sergio Babudieri[8], Laura A. Nicolini[9], Martina Milana[2], Pierluigi Cacciatore[4], Loredana Sarmati[3], Adriano Pellicelli[10], Stefania Paolucci[11], Antonio Craxì[12], Filomena Morisco[13], Valeria Pace Palitti[14], Massimo Siciliano[15], Nicola Coppola[16], Nerio Iapadre[17], Massimo Puoti[18], Giuliano Rizzardini[7], Gloria Taliani[6], Caterina Pasquazzi[5], Massimo Andreoni[3], Giustino Parruti[4], Mario Angelico[2], Carlo Federico Perno[19], Valeria Cento[1+*], Francesca Ceccherini-Silberstein[+1] on behalf of HCV Virology Italian Resistance Network (VIRONET-C)[$]

1. Experimental Medicine and Surgery, University of Rome "Tor Vergata", 00133 Rome, Italy. 2. Hepatology Unit, University Hospital of Rome “Tor Vergata”, 00133 Rome, Italy. 3. Infectious Diseases Unit, University Hospital of Rome “Tor Vergata”, 00133 Rome, Italy. 4. Infectious Diseases Unit, Pescara General Hospital, 65124 Pescara, Italy. 5. Infectious Diseases Unit, Sant’Andrea Hospital – “Sapienza” University, 00189 Rome, Italy. 6. Tropical Diseases, Umberto I Hospital –“Sapienza” University, 00161 Rome, Italy. 7. 1st Division of Infectious Diseases, ASST Fatebenefratelli Sacco, 20157 Milan, Italy. 8. Clinical and Experimental Medicine, University of Sassari, 07100 Sassari, Italy. 9. Infectious Diseases Unit, IRCCS AOU San Martino - IST, 16132 Genoa, Italy. 10. Hepatology Unit, San Camillo Forlanini Hospital, 00151 Rome, Italy. 11. Molecular Virology, Fondazione IRCCS Policlinico San Matteo, 27100 Pavia, Italy. 12. Gastroenterology, “P. Giaccone” University Hospital, 90127 Palermo, Italy. 13. Gastroenterology, «Federico II» University, 80131 Naples, Italy. 14. Hepatology Unit, Pescara General Hospital, 65124 Pescara, Italy. 15. Gastroenterology, Catholic University of Rome, 00168 Rome, Italy. 16. Infectious Diseases Unit, “L. Vanvitelli” University of Campania, 80138 Naples, Italy. 17. Infectious Diseases Unit, S. Salvatore Hospital, 67100 L'Aquila, Italy. 18. Infectious Diseases Unit, Niguarda Ca’ Granda Hospital, 20162 Milan, Italy. 19. Haematology and Oncohematology, University of Milan, 20122 Milan, Italy.

[+]V.C. and F.C-S contributed equally to this study.

***Corresponding author:** Valeria Cento, M.D., PhD, Department of Experimental Medicine and Surgery, University of Rome Tor Vergata; Via Montpellier 1, Rome 00133, Italy. Tel: +390672596560. Fax: +390672596039. E-mail: valeriacento@gmail.com

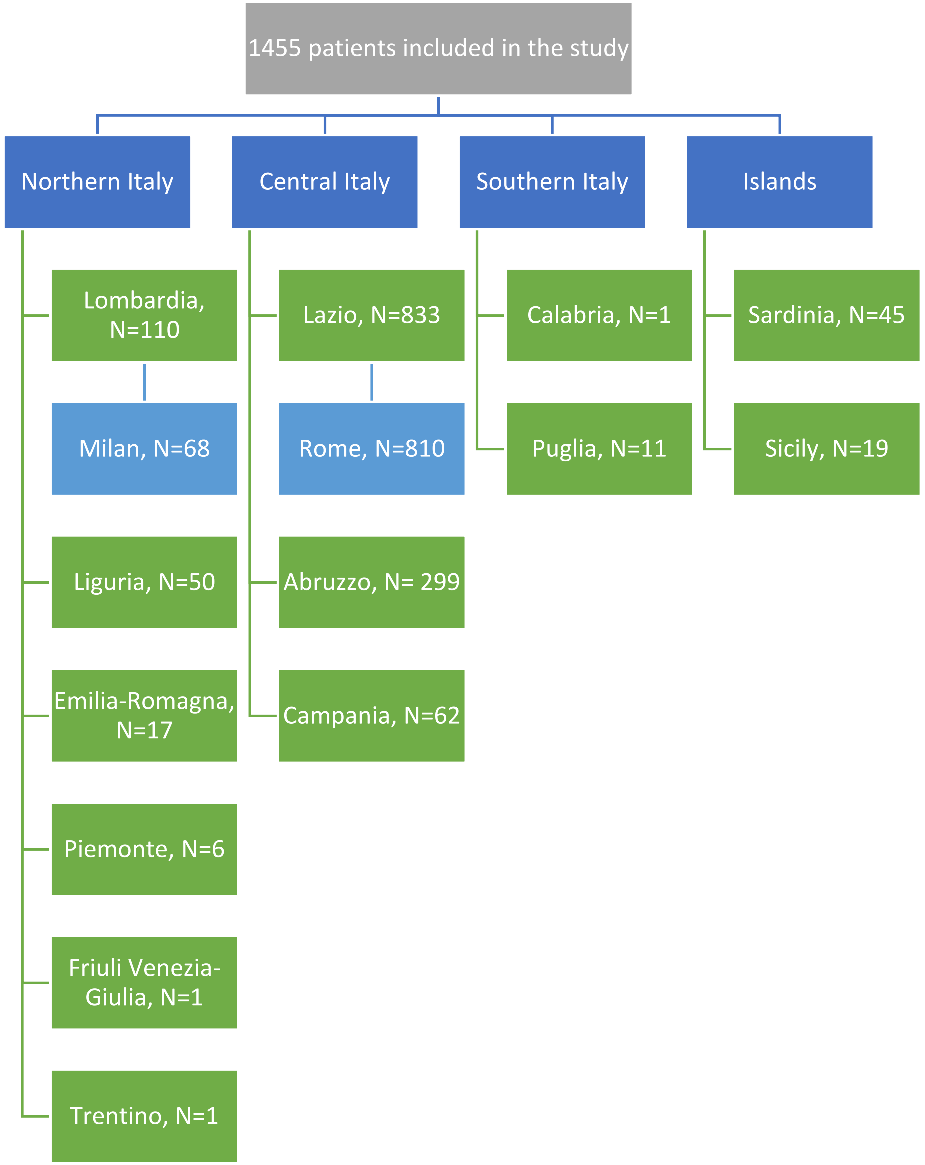

Supplementary Figure S1. Patients' enrollment among Italian regions. The number of patients enrolled in each Italian region is reported, along with focus on main cities as Milan and Rome.

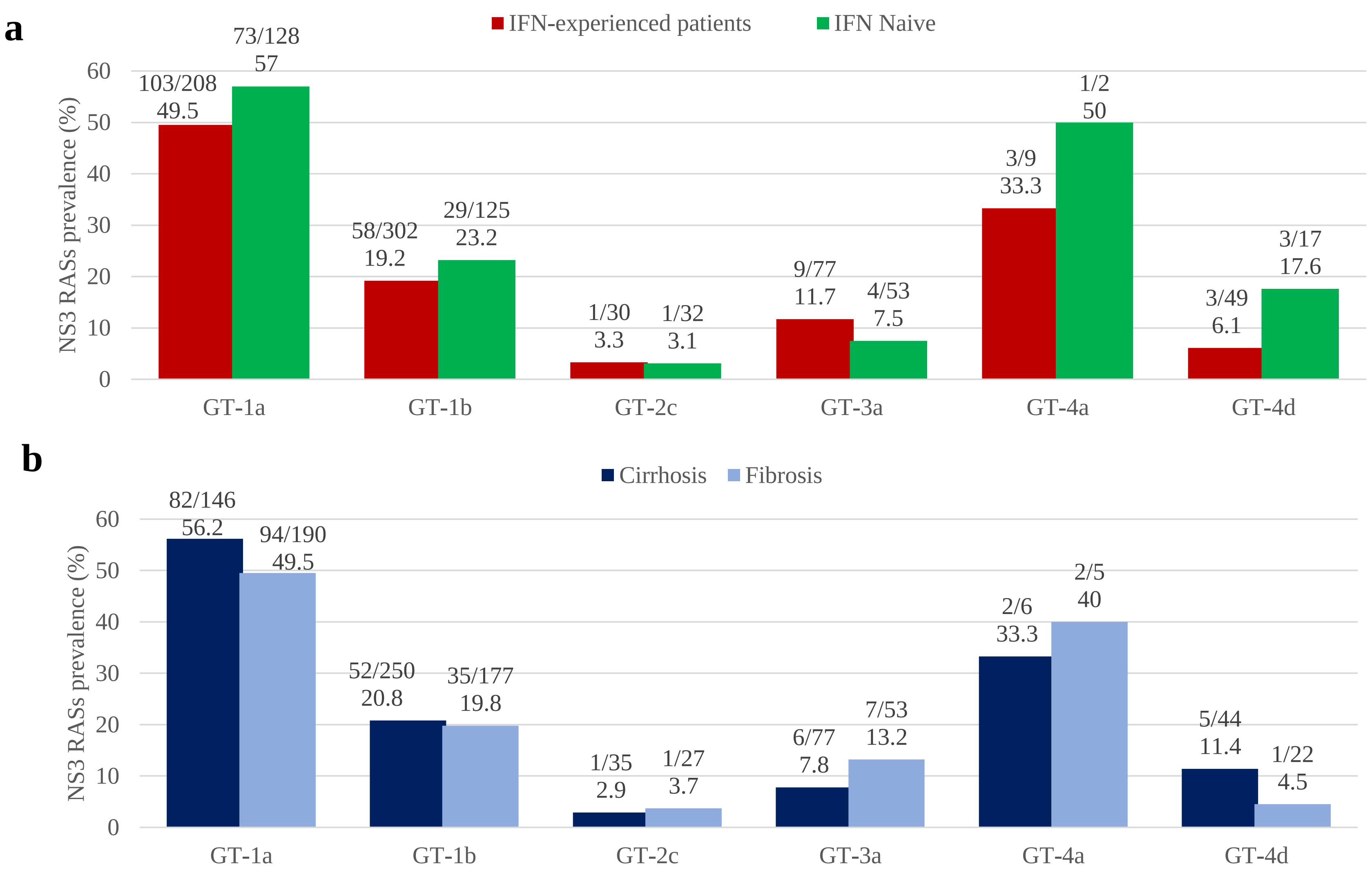

Supplementary Figure S2. Prevalence of natural NS3 RASs according to patients' characteristics and viral genotypes. NS3 RASs prevalence according to previous interferon treatment experience (**a**) or presence of cirrhosis (**b**) are reported. IFN, interferon; RASs, resistance associated substitutions.

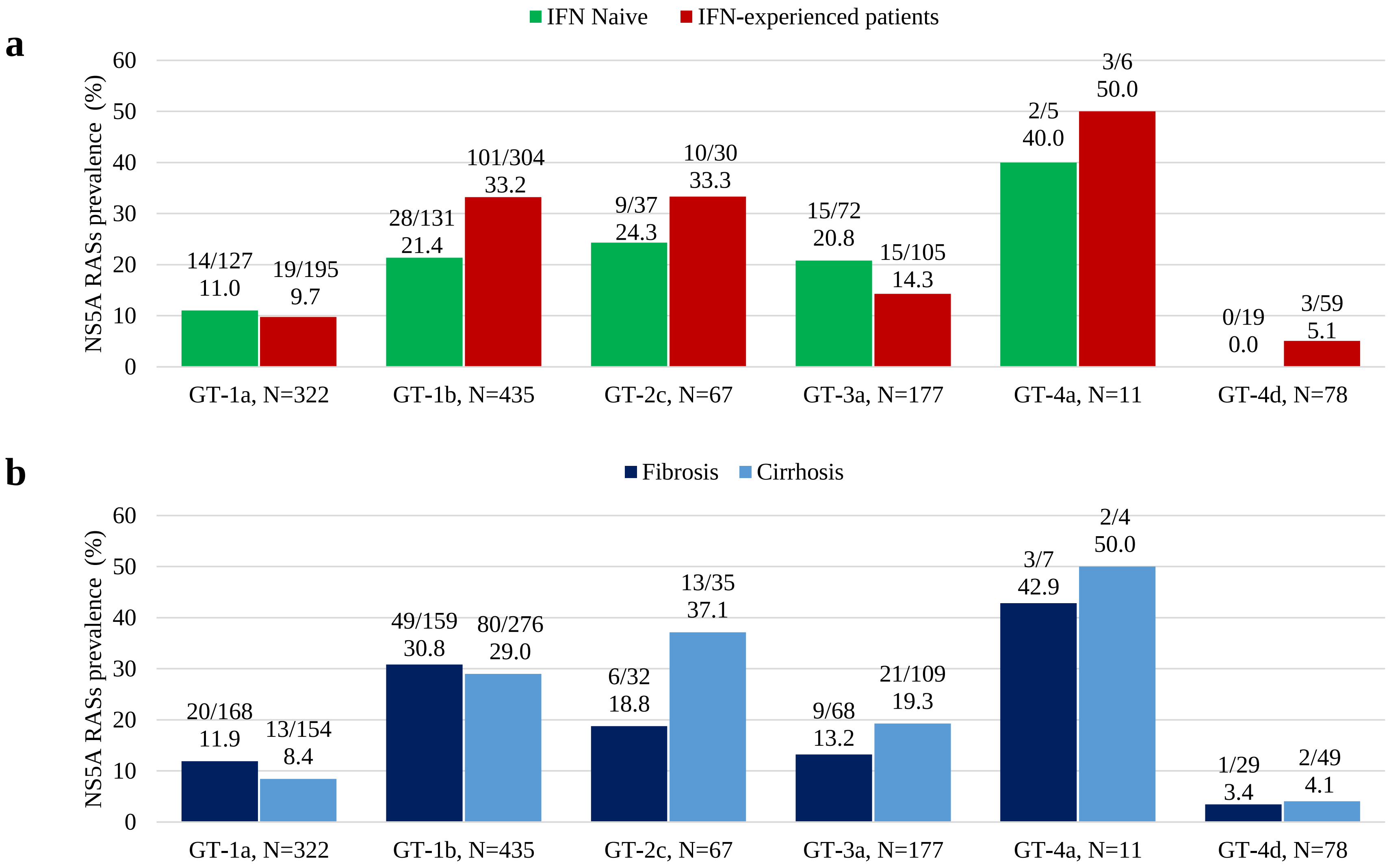

Supplementary Figure S3. Prevalence of natural NS5A RASs according to patients' characteristics and viral genotypes. NS5A RASs prevalence according to previous interferon treatment experience (**a**) or presence of cirrhosis (**b**) are reported. IFN, interferon; RASs, resistance associated substitutions.

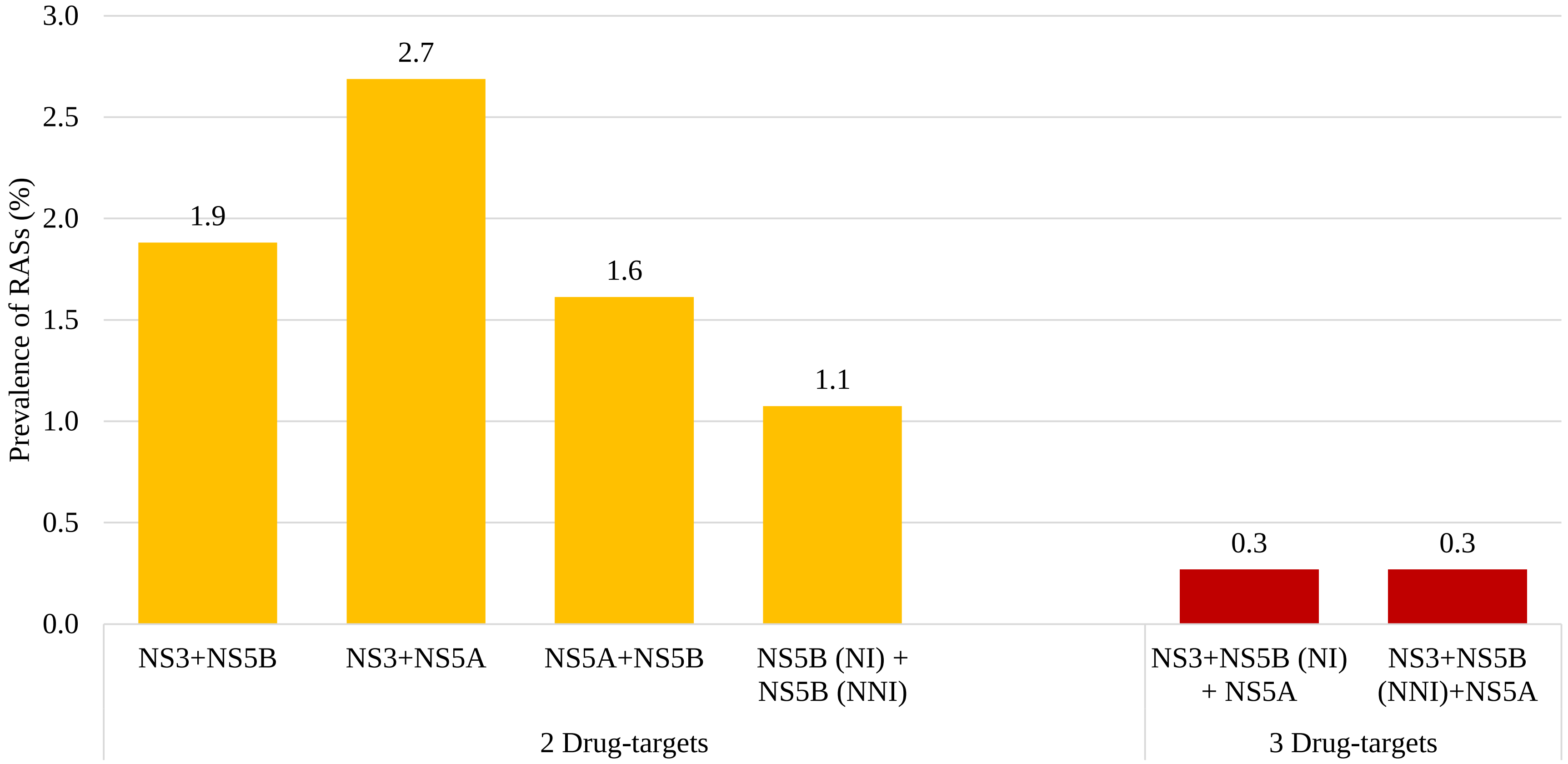

Supplementary Figure S4. Prevalence of multiclass resistance. Multiple RASs on 2 drug-classes are reported in yellow; multiple RASs on 3 drug-targets are in red. NS5B-NI, nucleoside NS5B polymerase inhibitors; NS5B-NNI, nonnucleoside NS5B polymerase inhibitors; RASs, resistance associated substitutions.

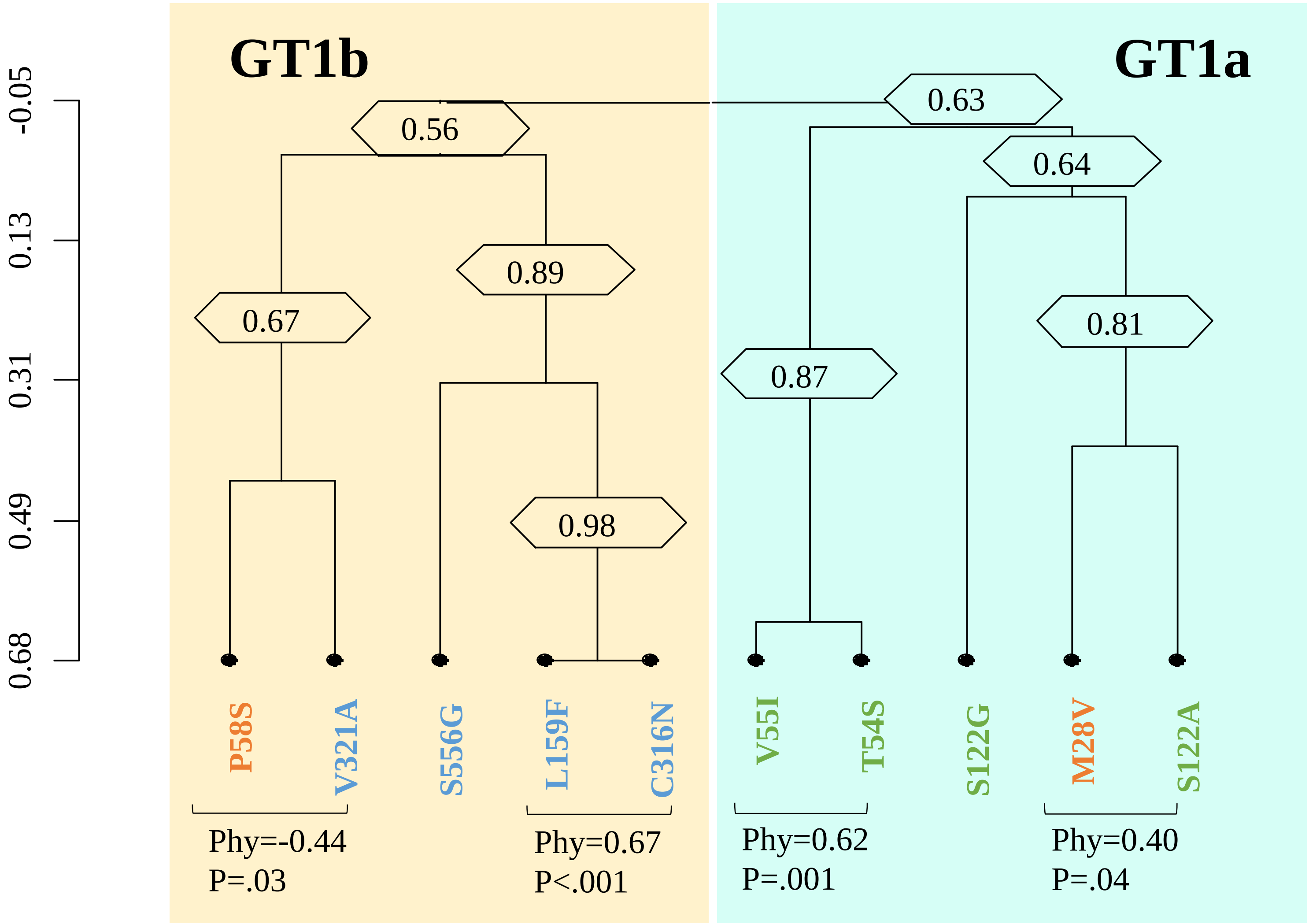

Supplementary Figure S5. Covariation analysis of natural NS3, NS5A and NS5B RASs.
NS3-RASs are reported in green, NS5A-RASs in orange, and NS5B-RASs in blue. Binomial-correlation coefficient (phi) was calculated to assess covariation among RASs, either on the same or on different genomic regions. Statistically significant pairs of RASs were identified by Fisher's exact test, and then corrected for multiple-testing by Benjamini–Hochberg method (FDR =.05). In order to identify and summarize higher-order interactions of RASs, we transformed the pairwise phi correlation coefficients into dissimilarity values. A dendrogram was then computed by hierarchical clustering, and its stability was assessed from 100 bootstrap replicates. All analyses were performed in R software. GT, genotype; RASs, resistance associated substitutions.
